# ‘Intern life’: a longitudinal study of burnout, empathy, and coping strategies used by French GPs in training

**DOI:** 10.3399/bjgpopen17X100773

**Published:** 2017-06-14

**Authors:** Eric Galam, Camille Vauloup Soupault, Lucie Bunge, Céline Buffel du Vaure, Emilie Boujut, Philippe Jaury

**Affiliations:** 1 GP, Département de Médecine Générale, Faculté de Médecine, Université Paris Diderot, Sorbonne Paris Cité, Paris, France; 2 GP, Département de Médecine Générale, Université Paris Descartes, Sorbonne Paris Cité, Faculté de Médecine, Paris, France; 3 GP, Département de Médecine Générale, Université Paris Diderot, Sorbonne Paris Cité, Faculté de Médecine, Paris, France; 4 Psychologist, Institute of Psychology, Paris Descartes University, Paris, France; 5 GP, Institut of Psychology, Paris Descartes University, Paris, France

**Keywords:** burnout, trainees, general practice, empathy, coping

## Abstract

**Background:**

More than half of French medical GP trainees (GPTs) suffer from burnout.

**Aim:**

To define and follow the evolution of risk factors, such as empathy and coping strategies, associated with burnout in this population.

**Design & setting:**

Prospective longitudinal study involving volunteers of 577 Parisian university GPTs in 2012.

**Method:**

Self-reported anonymous online questionnaires were sent three times every 6 months to all participants. Stress was measured using the Intern-Life scale and burnout using the Maslach Inventory, and anxiety and depression measured using the Hospital Anxiety and Depression Scale (HADS). Sociodemographic, professional, and personal data, including coping strategies and measures of empathy were also collected.

**Results:**

In total 343 questionnaires were fully completed at baseline (T0): 304 were usable at baseline, 169 were usable at 6 months (T1) and 174 at 1 year (T2). Stress rates decreased sharply between T1 (scores 42.96) and T2 (17.08), while scores for burnout remained relatively stable: more than 13% of GPTs had high scores in all three dimensions of burnout. Depersonalisation increased from 61% (T1) to 66% (T2). One hundred and four paired samples were analysed between T0 and T1, and between T1 and T2. Emotion-centred coping was associated with emotional exhaustion (*P*<0.05), while professional support reduced it. Experiences of aggression increased depersonalisation (*P*<0.05). Social support, problem-centred coping, perspective-taking empathy, and professional support improved the sense of personal accomplishment (*P*<0.05).

**Conclusion:**

Tools to help GPTs are available but are underused. More training in doctor–patient relationships and understanding of medical hidden curricula are necessary to decrease burnout among GPTs and improve their wellbeing and patient care.

## How this fits in

Burnout is described with increasing frequency among French doctors and trainees, especially among GPs.

The links between empathy and professional characteristics have been studied in France among GPs and GPTs but no longitudinal study has ever been conducted.

This is the first French longitudinal study of GPTs over their first year in training, and confirms a high prevalence of burnout, as well as the increasing depersonalisation of doctors and the importance of some risk factors including ways of coping as a caregiver and of being empathic to patients, and the impact of the hidden medical curriculum.

## Introduction

According to Maslach and Leiter:^1^

‘… burnout is a psychological syndrome emerging as a prolonged response to chronic interpersonal stressors on the job. The three key dimensions of this response are an overwhelming exhaustion, feelings of cynicism and detachment from the job, accompanied by a sense of ineffectiveness and lack of accomplishment. The significance of this three-dimensional model is that it clearly places the individual stress experience within a social context and involves the person’s conception of both self and others.’

Widely described in the medical world, burnout is frequent among French GPs, and other doctors.^2–5^ It is also frequent among medical trainees in general practices (GPTs).^6–9^ Its consequences can be dramatic^10^ and its causes complex.^11–16^ Several multifactorial explanations have been proposed, including links with stress,^17–20^ depression,^21^ sleep deprivation,^22^ and medical error.^2,23^

Three aspects of this problem are particularly important. First, ways of understanding others through empathy,^24–27^ which reflects the ability of the caregiver to understand the point of view of his or her client. Secondly, managing one’s own problems by coping,^17,28–29^ which is the ability to overcome the impact of an event perceived as threatening. It can be based on three categories of strategy centred on a) the problem, b) the seeking of social support, and c) the emotions entailed. Third, the ‘hidden curriculum’,^30^ which acknowledges that medical education is more than the simple transmission of knowledge and skills; it is also a process of ‘socialisation’, including norms and values about how to be a good doctor. The main objective of this study was to explore possible predictors in the evolution of burnout among Parisian GPTs during their first year internship, and to assess their correlation to empathy and coping, and to highlight the role of the hidden curriculum (Box 1).^31^

**Box 1.** French GP training course

After 6 years of medical studies, each French medical trainee has to choose a specialisation. A French medical student studies 3 years to specialise as a GP. This includes, at least: 200 hours of theoretical coursework. Six practical courses of 6 months in medical settings including one in an outpatient general medicine setting, one in emergency, one in internal medicine and one in gynecology or paediatrics, and two other specialties of the trainee’s choice. GPTs have a biannual meeting to choose their next 6-month placement. GP trainees were offered participation in this study at these meetings. Proof of learning as evidenced by written stories anecdotes of an experience as a practitioner including their analysis of authentic clinical situations. Thesis on a topic for a GP.

## Method

### Participants and setting

This work is a part of the ‘Intern life study’, a 3-year follow-up study of a cohort of Parisian GPTs, which started in October 2012.

Participants were all general practice residents in their first year training. There were no exclusion criteria. The study was presented by GPTs during their first meeting where residents-in-training choose the topic and location of internship every 6 months. Five gifts were drawn (free cinema tickets) to encourage participation. Anonymous online questionnaires were sent with reminders to participants at three different times: at baseline (T0), after 6 months (T1), and after 1 year (T2). A free telephone number was provided to help GPTs seeking help for themselves if necessary.

### Measures

This study collected extensive longitudinal data on the GPTs, some of which is reported elsewhere.^32–34^ The results presented here are about burnout and empathy, at the T0 timepoint (baseline), T1 (6 months) and T2 (October 2013).

Before use, the questionnaire (available from authors on request) was tested on three GPTs. It contained 53 questions and took 20 minutes to complete. In addition to demographic, work, environmental, and behavioural data, it explored levels of burnout, depression, stress, and transactional variables such as coping strategies and clinical empathy toward patients.

The following validated questionnaires were used:

- burnout measured by the Maslach Burn-out Inventory (MBI);^1^
- empathy by the Jefferson Physician Empathy Scale (JSPE scale);^35^
- coping strategies by the Ways of Coping Checklist-Revised Questionnaire (WCC-R);^36^
- stress by a specific stress questionnaire for GPTs developed and validated by the qualitative part of the ‘Intern-life’ study;^37^
- anxiety and depression by the HADS;^38^ and
- other variables: doctor–patient training, professional support, current training, workload, aggression towards the doctor and conscientiousness.

### Statistical analysis

Descriptive univariate analyses were complemented by bivariate analysis using Spearman and Pearson tests between burnout dimensions and quantitative variables. Qualitative variables were explored using Student *t* tests. The variables associated in the first part of the study with *P*<0.01 were selected for multivariate analysis by logistic regression.

Analyses on paired samples were conducted, averaging the scores by a same participant at T0 and T1, and then at T1 and T2. Student *t* tests were used to compare the average of repeated measurements to track the evolution over time of burnout and other variables. The effect of variables on the three dimensions of burnout were compared by analysis of variance for repeated measures (ANOVA). A *P-*value of <0.05 was considered significant. Statistical tests were performed with the SPSS 17.0 software.

## Results

### Population characteristics

**Figure 1.**
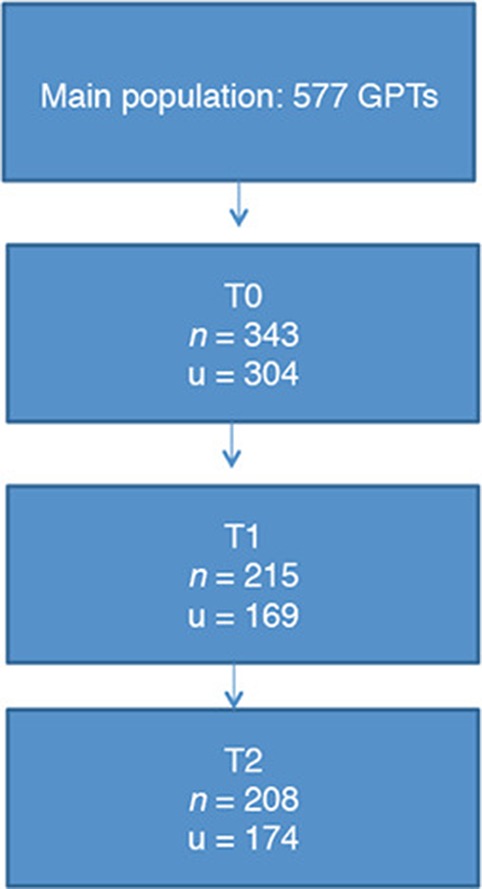
Flowchart of the study. Number of GPTs at each time. n = number of answers. u = number of utilisable questionnaires.

Among all 577 Parisian GPTs starting their internship, 304 questionnaires were complete and usable at T0 (response rate: 52.6%), 169 (29.3%) at T1, and 174 (30.2%) at T2. At the three time points, an average of 76.1% of GPTs were female, with an average age of 25.4 years (range 22–43 years). See Table 1 and Figure 1.

**Table 1.** Population at the three study times

	T0 (*n* = 304) before starting course	T1 (*n* = 169) after 6 months	T2 (*n* = 174) after 12 months
	*n* (%)	*n* (%)	*n* (%)
**Demographics**			
Female	258 (75.2)	166 (77.2)	158 (76.0)
Male	85 (24.8)	49 (22.8)	50 (24.0)
Living alone	167 (48.7)	99 (46.0)	79 (38.0)
**Location of rotation**			
Accident & Emergency		87 (40.5)	97 (46.6)
Internal medicine		102 (47.4)	88 (42.3)
Paediatrics		9 (4.2)	8 (3.8)
Gynaecology		3 (1.4)	2 (1.0)
Private GP practice		1 (0.5)	8 (3.8)
Free course		13 (6.0)	5 (2.4)
**Job characteristics**			
Satisfaction rating course, 1–10		7 (1.9)	7 (1.7)
Working hours per week, *n*		56 (16.9)	59 (38.4)
Duty nights per month, *n*		3 (2.0)	3 (2.2)
Free weekends per month, *n*		2 (1.1)	2 (0.8)
Free weeks per 6 month course, *n*		2 (0.9)	4 (0.9)
Transport time per day, minutes		88 (51.5)	84 (45.2)
**Professional experiences**			
Moral harrassment		11 (5.1)	16 (7.7)
Sexual harrassment		3 (1.4)	5 (2.4)
Conscientiousness-hitting		138 (64.2)	128 (61.5)
Aggression		74 (34.4)	89 (42.8)
Clinical part of work		169 (64.2)	174 (64.5)
Administrative part of work		169 (27.7)	174 (28.3)
Professional support		176 (81.6)	177 (85.1)
Doctor–patient relationship training		151 (70.2)	75 (36.1)
Predominance of informal training compared to formal training		191 (91.0)	189 (94.0)
Positive scientific role-modeling doctors		196 (93.3)	193 (96.0)
Compassionate role-modeling doctors		199 (94.8)	184 (91.5)
Negative scientific role-modeling doctors		147 (70.0)	146 (72.6)
Non-compassionate role-modeling doctors		159 (75.7)	145 (72.1)

### Univariate analysis at T1 and T2

#### Burnout scores

At T1, 61% had at least one dimension of high burnout and more than 13% had high scores in the three dimensions. At T2, these figures remained almost stable at 66% (one dimension) and 13% (three high dimensions). Emotional exhaustion rates were 46% at T1 and 47% at T2. Depersonalisation rate was 61% at T1 and 66% at T2; and low personal accomplishment rates were 34% at T1 and 35% at T2 (Table 2).

**Table 2.** Burnout scores

	T1, %	T2, %
Three high scores	14	13
Two high scores	17	13
One high score	30	40
No high score	39	34
High emotional exhaustion	46	47
High depersonalisation	61	66
Low personal accomplishment	34	35

#### Empathy

A T1 and T2, the three dimensions of empathy were distributed similarly across the study population (Table 3).

**Table 3.** Empathy and coping strategies at T1 and T2

	Mean		Median		Standard Deviation	
	T1	T2	T1	T2	T1	T2
**Empathy**					*n* = 197	*n* = 197
Taking perspective	50.3	51.9	50	52	7.9	7.9
Compassionnate care	43.7	43.5	44	44	5.8	6.1
Put in the patient’s place (seeing things from the patient’s point of view)	9.8	10.1	10	10	2.4	2.3
						
**Coping**					*n =* 172	*n =* 179
Problem centred	26.7	27.6	27	28	6.4	6.4
Support centred	20.8	21.7	21	22	4.6	4.4
Emotion centred	21.7	21.9	22	22	6.4	5.8

#### Anxiety

At T1, the average anxiety score was 11.21 (standard deviation [SD] 2.38) showing anxiety for more than 50% of GPTs (197 responders). It remained stable at T2 with a score of 11.02 (SD 2.183).

#### Depression

At T1, the average depression score was 8.18 (SD 1.649): most GPTs had no evidence of depression scores. It remained stable at T2 with 8.39 (SD 1.684).

#### Coping strategies

At T1 and T2, the three coping strategies were distributed similarly across the population (Table 3).

#### Stress

A T1, GPTs had an average stress score of 42.96 with a median of 44 and an SD of 9.58 for 169 responders. At T2, the stress rate was much lower at 17.08 (SD = 4.05) for 174 responders.

### Bivariate cross-sectional analysis

A T1, emotion-centred coping strategies appeared to be positively associated with the three burnout dimensions.

At T1 and T2, being assaulted had a significant impact on emotional exhaustion and depersonalisation (*P*<0.01). Conscientiousness was correlated with depersonalisation at T1 and with emotional exhausation at T2.

At T1 and T2, GPTs who had received training on doctor–patient relationships were less likely to experience emotional exhaustion (Box 2).

**Box 2.** Main results on paired samples *n* = 104 (for the same persons at two different points)

	Transversal analysis		Longitudinal analysis	
	Positive correlations	Negative correlations	Positive correlations	Negative correlations
**Emotional empathy**	No correlation	Stress (T1 and T2) Emotion-centred coping^a^ (T1, T2) Anxiety (T1) Moral harrassment (T2) Conscience-hitting (T2)	Professional support	Stress Emotion-centred coping^a^
**Depersonalisation**	No correlation	No correlation	No correlation	Aggression
**Low personal accomplishment**	Emotion-centred coping (T1, T2)	Problem-centred coping^a^ (T1, T2) Taking perspective empathy (T1, T2)^a^ Dining out (T1 and T2)		Social-support centred coping Problem-centred coping^a^ Taking perspective empathy^a^ Professional support

^a^Correlations found in all study analysis.

### Multivariate cross-sectional analysis

#### Factors associated with emotional exhaustion

Stress at T1 (odds ratio [OR] 1.07; 95% confidence interval [CI] = 1.02 to 1.13) and at T2 (OR 1.2; 95% CI = 1.03 to 1.32), emotion-centred coping (OR 1.1; 95% CI =1.01 to 1.22) at T1 and at T2 (OR 1.14; 95% CI = 1.05 to 1.23) were statistically associated with emotional exhaustion.

At T1, there was also an association with anxiety (OR 1.3; 95% CI = 1.15 to 1.41) and at T2 with ‘conscientiousness-hitting’ — that is, being unable to live up to one’s personal values — (OR 1.34; 95% CI = 1.15 to 1.79).

At T1 and T2, no significant impact was found with the number of working hours, satisfaction with the course, professional support, doctor–patient training, and aggression towards the doctor.

#### Factors associated with depersonalisation

No variable was significantly associated with depersonalisation at T1 or T2.

#### Factors associated with low personal accomplishment

Strong emotion-centred coping rate was associated with low personal accomplishment (OR 1.11; 95% CI = 1.01 to 1.22) at T1 and at T2. Conversely, a high problem-centred coping rate (OR 0.86; 95% CI = 0.77 to 0.96) and taking perspective empathy (OR 0.92; 95% CI = 0.86 to 0.98) were associated with a high rate of personal accomplishment).

### Longitudinal analysis of paired samples

#### Average comparison test

Among the 104 analysed subjects, significant changes for a particular person across two time points (T0 and T1, and T1 and T2) were:

- three dimensions of burnout increased significantly (*P*<0.01);
- taking perspective empathy decreased (*P* = 0.06);
- leisure and clinical (compassionate) empathy decreased significantly *P* = 0.027 (leisure) *P* = 0.016 (empathy); and
- anxiety increased (*P*<0.01)

### Analysis of variance of repeated measures (ANOVA)

**Figure 2.**
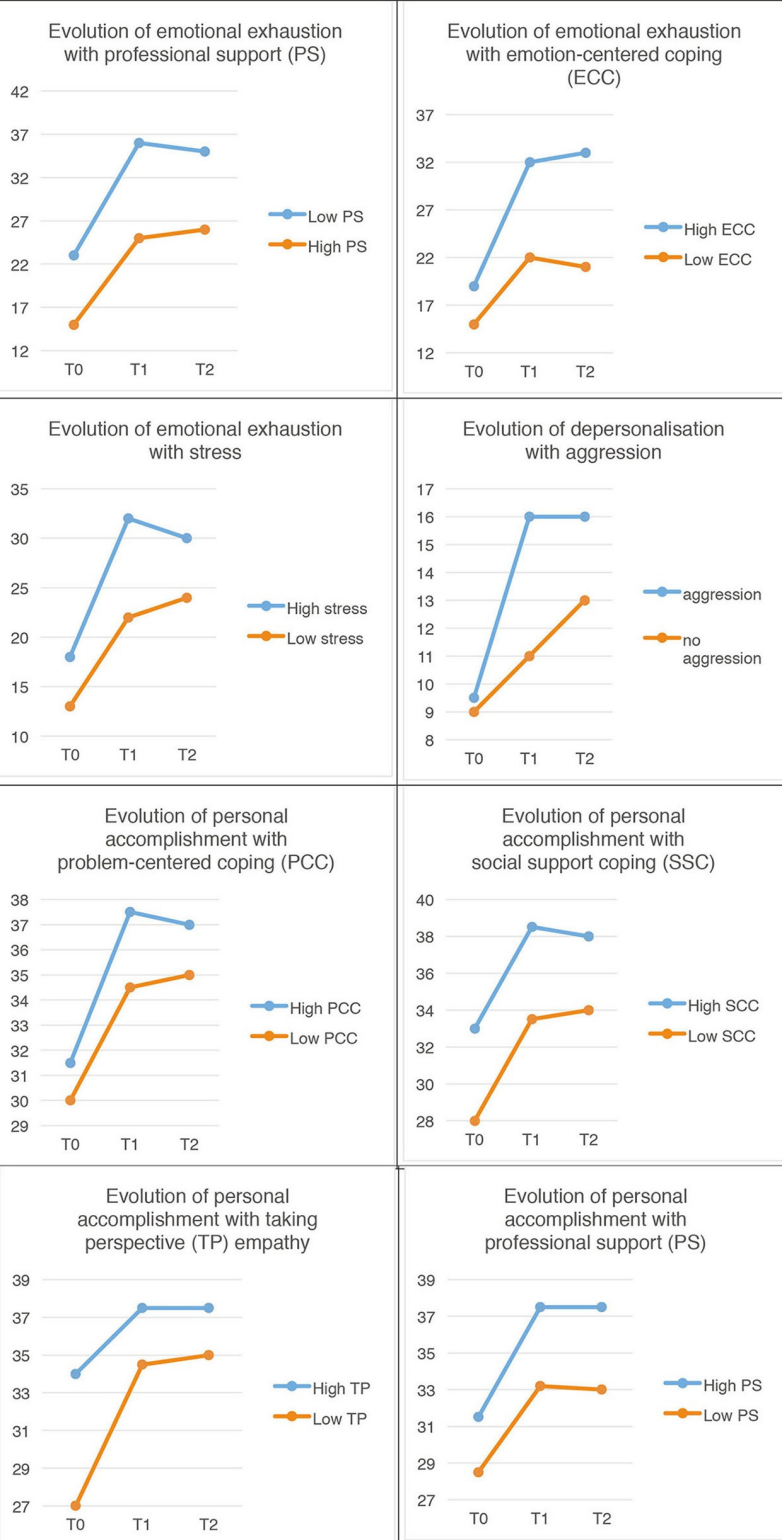
Evolution of correlations during the study for 104 paired samples.

An increase in emotional exhaustion (Figure 2) was associated with emotion-centred coping (*P*<0.01) and stress (*P* = 0.02). A decrease in emotional exhaustion was associated with professional support (*P*<0.01). An increase in depersonalisation was associated with being assaulted (*P* = 0.02). An increase in personal accomplishment was associated with problem-centred coping (*P* = 0.05), social-support centred coping (*P*<0.001), empathy (*P*<0.0041), and professional support (*P*<0.0304).

## Discussion

### Summary

This is the first major longitudinal study to define and follow the evolution of risk factors, inclusion empathy and coping strategies, associated with burnout in French GP trainees. Positive findings included stress rates decreasing sharply during the study period and overall low scores for anxiety and depression on the HADS. However, levels of burnout, particularly depersonalisation, were high. In paired, within-subject analyses, emotion-centred coping strategies contributed to, and professional support protected against, emotional exhaustion. Aggressiveness towards doctors increased feelings of depersonalisation. Trainees’ sense of personal accomplishment improved through social support, problem-centred coping, empathy and professional support.

### Strengths and limitations

There have been no other longitudinal studies on burnout in French GPTs. The entire questionnaire used validated tools and in addition to individual and professional risk factors, interpersonal factors were explored.

This study has a few limitations. There was a selection bias: the average age and personal status of GPTs in the current study are similar to other French studies,^6,9,39^ and the participants are all living in the Paris region. Therefore, it is difficult to generalise these findings to all French GPTs. Also, the questionnaire was self-completed via the internet, and did not allow for the control of the amount of time for completion or the assurance that only one questionnaire was filled out per participant.

There was also a measurement bias: there are many definitions of burnout and although the MBI is validated and widely used, allowing for comparisons with other findings, it does not distinguish between formal, potential, or obvious burnout. This study looked for factors associated with the severe stages of each of the three dimensions of burnout and did not integrate moderate scores, which are regarded as pathological by some authors. The method of self-questionnaire necessarily produced subjective responses and may be influenced by social desirability bias or modesty. Finally, grids, whatever their validity criteria, are all reductive, especially when they explore experience in a declarative way.

### Comparison with existing literature

Almost 32% of all GPTs participated in this study at T1 and T2. It decreased from the beginning rate (T0) but it is of note that participants at T0 had not yet begun their training. This rate is lower than comparable studies on GP or GPT burnout where rates are >60%.^6,8^ However, it is important to note that the questionnaire took 20 minutes to complete and was required to be repeated three times.

The burnout scores were much higher than in the literature. Results showed a medium emotional exhaustion score of 27.6 versus 19.5 in subsequent thesis work.^3^ The depersonalisation scores were 19.9 versus 10 and low personal accomplishment at 36 versus 37.4 in the literature.^6^ This can be explained by the fact that this study population concerned young interns in their first or second semester. This assumption confirms Barbarin’s work,^40^ showing that 70% of first year GPTs meet at least one of the high burnout criteria (versus 64% in the current study).

Concerning social life and leisure, this study found similar results to those in the literature.^7–8,41^ The time devoted to private life seemed to be protective for burnout, as well as increasing personal accomplishment

At T1 and T2, no significant association was found with the number of working hours, satisfaction with the training course, professional support, doctor–patient training, and aggression. This is hard to believe, but confirms other studies^7^ that found other risk factors including a poorly defined role, lack of support, difficulties in expressing one’s doubts, disproportionate responsibilities, discrimination, harassment, the gap between business expectations and reality field, or violence. This study confirms this direction by finding that professional support as protective for burnout and ‘conscientiousness hitting’ — being unable to live up to one’s professional values — the experience of aggression, and stress are risk factors for burnout.

The study showed that ‘taking perspective’ empathy is protective for burnout at T1 and T2 and in the longitudinal analysis. As in the literature,^42^ clinical (compassionate) empathy decreased over the course of the GP internship in the current study.

A multicentre study conducted in 2004 with all medical students of Minnesota correlated the progressive loss of empathy with emotionally difficult experiences of the student during their course of hospital work, and poor quality of life, unlike feelings of wellbeing that were correlated with a high degree of empathy.^18^

If empathy proves to be a powerful help in the understanding of patient needs, it is altered during the internship. Emotion-centred coping is associated with emotional exhaustion and a decrease in personal accomplishment. Active coping strategies (problem-centred and social support-centred strategies) decreased burnout. Emotion-centred coping was found to be an avoidance strategy that does not solve the problem and can cause stress.

### Implications for research and practice

A number of interventions have been introduced in France to treat burnout in GPs and GPTs within hospital services.^43^

Like other doctors, GPTs are often overloaded and continue working despite blatant warning signs.^44^ They need to be able to accept their weaknesses and to ask for help if necessary.

Here, organisational aspects join cultural expectations. Like any other social activity, basic rules should be respected within medical practice. For example, the compensatory rest^45^ after being on call (as detailed in a recently proposed law^46^ limiting GPs working time to 48 hours per week), is far from being fully implemented. External stressors could be studied to see how systemic/policy change can be implemented to minimise their contribution to burnout. Qualitative studies can also help to understand and take the necessary actions for the development of medical education and GPTs development.

Cultural aspects converge, explain, and strengthen social aspects. Becoming a doctor is not only founded on knowledge and practice, but also on acculturation to a more or less dogmatic conception of medical practice. This ‘hidden curriculum’^47^ requires the trainee doctor to absorb and bend to the implicit, yet pervasive, standards of the medical community: no complaints or even criticism from their superiors, no emotional or personal disease states, especially if they are related to the practice of medicine. As such, burnout, even more than non-psychological diseases such as heart failure or cancer, is marked as a transgression, or of infamy and treason for the medical establishment. Managing uncertainty and difficulties, dealing with medical errors when they occur, overcoming the loss of meaning and negotiating ethical dilemmas, treating relatives and one’s own diseases, treating other sick doctors … all these situations are, with few exceptions,^48^ not formally addressed in the medical curriculum, as if they did not exist or as if everyone should manage them alone, as a personal problem to be hidden.

This amplifies the fact that training for managing in patient–doctor relationships is still considered as implicit and comprises only a very small part of the formal curriculum. In addition to the overworking,^49^ medical practice sometimes leads to humiliation and a lack of sensitisation for GPs.^50^

Beyond the organisation and resources of the healthcare system and those of each caregiver, taking care of oneself and protecting one’s social life is essential,^14^ since it has been shown that individuals who have many resources, as they are often less sensitive to stress perception, use active coping, and have less risk of burnout.^15^

## Authorisation

Authorisation was given by the French National Commission for Data Security (CNIL).
